# Polymorphisms in Tunisian patients with N-acetylgalactosamine-6-sulfate sulfatase gene deficiency: Implication in Morquio A disease

**DOI:** 10.1186/1746-1596-6-11

**Published:** 2011-01-20

**Authors:** Souhir Khedhiri, Latifa Chkioua, Salima Ferchichi, Abdelhedi Miled, Sandrine Laradi

**Affiliations:** 1 Laboratory of Biochemistry CHU Farhat Hached, Sousse, Tunisia; 2 Laboratory of Biochemistry and Molecular Biology, Monastir, Tunisia

## Abstract

Mucopolysaccharidosis type IVA or Morquio A syndrome is characterized by the lack of N-acetylgalactosamine-6-sulfate-sulfatase and the accumulation of keratan sulfate and chondroitin-6-sulfate in the lysosomes. At least, 148 mutations and 16 polymorphisms were identified in the GALNS gene.

The aim of this study was the screening of polymorphisms within 7 patients recruited from many regions of Tunisia in order to determine the haplotypes and their association with the mutations previously reported.

**Patients and methods:**

We have used the PCR sequencing to analyse the different haplotypes and to identify the polymorphisms within 7 affected MPS IVA patients.

**Results:**

Nine GALNS polymorphisms were detected in the 7 studied patients. Five of these polymorphisms are within the GALNS gene exons. Six polymorphisms have been previously described and used for linkage analysis in MPS IVA patients and determination of haplotypes. We have identified two novel heterozygous polymorphisms in intron 13 and intron 3

**Conclusion:**

Polymorphisms may be useful for carrier detection and prenatal diagnosis in informative families whose specific mutations have not been identified. The determination of haplotypes can also determine the origin of some mutations in a population.

## Background

Mucopolysaccharidosis IVA (Morquio A syndrome; MPS IVA) is an autosomal recessive disease that is classified in the group of mucopolysaccharide storage diseases. It results from a deficiency of the lysosomal enzyme N-acetylgalactosamine-6-sulfate sulfatase (GALNS; E.C.3.1.6.4) [1], which hydrolyses N-acetylgalactosamine-6-sulfate and galactose-6-sulfate in glycosaminoglycans. GALNS deficiency causes intralysosomal accumulation and urinary secretion of undegraded keratane sulfate (KS) and chondroitine-6-sulfate (C6S).

Phenotypes in Morquio A disease vary from the classical form with severe bone dysplasia, heart valve involvement, corneal opacity, short trunk dwarfism and a life span of 20 to 30 years, to attenuated forms with normal life span, mild bone involvement and mild visceral organ involvement. Unlike the other forms of mucopolysaccharidoses, Morquio A disease is characterized by normal intelligence.

Investigations of the allelic heterogeneity in MPS IVA have been facilitated by the isolation and characterization of the full length cDNA encoding the human GALNS protein and the genomic GALNS gene [2]. The GALNS gene spans 35 kb in length, contains 14 exons and 13 introns, and was mapped to the region 16q24, by in situ hybridization [3]. To date, multiple mutations responsible for various clinical phenotypes of MPS IVA have been described [4]. Sixteen polymorphisms have been also detected in the GALNS gene [5-7]. Some polymorphisms were used for the haplotype analysis.

In the present paper, we have screened the GALNS gene of 7 affected MPS IVA patients using genomic DNA samples and performing polymerase chain reaction (PCR) in combination with DNA sequence analysis.

## Patients and methods

### Patients

The study was carried out on 7 MPS IVA patients recruited in paediatric departments of different geographic areas of Tunisia (Central and Southern areas). It was approved by the Ethics Committees of the respective Tunisian hospitals, and the families gave informed consent before withdrawal of blood.

Family and history description of the 7 studied patients were summarized in table 1.

**Table 1:** Description of the 7 MPS IVA patients yr: years, mo: months

Features	Patient 1	Patient 2	Patient 3	Patient 4	Patient 5	Patient 6	Patient 7
Consanguinity of the parents/degree	1st degree	1st degree	3rd degree	3rd degree	1st degree	1st degree	1st degree

Age of diagnosis (yr/mo)	3	8	3	5/4	4/5	7/7	7

Age of onset (yr/mo)	18 months	14 months	2	16 months	2/3	18 mo	3

Sex	Male	Female	Male	Female	Female	Female	Female

Age (yr/mo)	15	13	9/8	8	11/7	Died at 7yr	14

Height cm (SD)	1 m (-4.8)	96 (-6)	95 (-5.9)	90 (-6.6)	96 (-8.4)	98 (-5)	122 (-4)

Weight kg (SD)	17 (-3.2)	14 (-3.3)	16 (-3.1)	15 (-2.3)	16 (-3)	15 (-2.3)	26 (-2.3)

GALNS assay (nmoles/h/mg prot)	0.04	0.02	0.03	Not done	0.005	0.06	0.07

The patients were always consanguineous but the families completely unrelated to each other and lived far apart. All the affected children have healthy sibling(s). Patients 1 - 2 and 3 - 4 belong to the same siblings.

#### Patients 1

He was the second child of healthy first degree consanguineous parents originated from the South of Tunisia and no skeletal disorders had occurred in the family. The patient was delivered vaginally after an uncomplicated full-term pregnancy. He was eutrophic at birth and during the first year of life, psychomotor and mental characteristics were perfectly normal. From 18 months of age, he had progressive walking troubles with tendency to fall. At the age of 3 years, he has been operated for inguinal hernia. The surgeon revealed osseous malformations such as genu valgum, dorsal and lumbar cyphosis. At the age of 9 years, he wore an orthopedic corset without any result. Marked bone dysplasias (sternal bulge, disproportional dwarfism with short trunk and neck) with arthrochalasis associated with functional troubles and facial dysmorphism (peg shaped dentition, head rejection) led to the diagnosis of Morquio A syndrome.

Radiologic examination detected skeleton malformations on cervical rachis (flat cervical vertebra, hypoplasia of the odontoid apophysic second vertebrae), on thorax (oar-appearing ribs) and rachis. The ophtalmological examination was normal. Morquio A syndrome was based on findings of keratansulfaturia and very low level of GALNS activity in leukocytes. At the age of 15 years, the examination of the patient highlighted important osseous deformations and abdominal distension without spanchnomegalia. The neurological examination was normal. He was doing excellently in the high school, especially in maths and physics although many practical problems such as speed of writing, transportation as he still could barely walk. During the survey, we could ask for an electric handicapped adapted car to allow him to go to school easier. The patient's phenotype was classical Morquio A disease, severe form.

#### Patient 2

This girl of 2 years, the sibling case of patient 1 was asymptomatic when MPS IVA was confirmed by enzymatic assay with a very low GALNS activity. The vaginal delivery and the postnatal period were without any complication. Since then, the patient has developed gradually scoliosis, pectus carinatum and severe valgus deformities. She was intellectually normal but had quickly decreased motor function and barely walked from the age of 5 years, and finally could not go to school, contrary to her brother. One year later, a spastic paraplegia occurred. Cervical MRI showed a cervical spine compression. After performing surgery stabilizing the upper cervical spine, she had partially recovered.

#### Patient 3

Patient 3 appeared perfectly normal at birth after vaginal delivery. His father was married with his cousin's daughter. At the age of 3 years, the patient had a waddling gate with a tendency to fall, that required medical studies at paediatric unit of Kairouan hospital (central Tunisia). Marked growth retardation, facial dysmorphism with a particular protrusion, bone dysplasias, short trunk and neck, sternal bulge, disproportional dwarfism led to the clinical diagnosis of Morquio syndrome. Keratane sulfaturia was detected and enzyme assay revealed MPS IVA. At the age of 8 years, further skeletal X-rays investigation typically showed marked flattening of the vertebra, oar-appearing ribs, cyphosis with generalized platyspondylia. Cardiac examination detected and left ventricular hypertrophy.

Neurological investigation was normal in spite a relative retardation in school performances which may be explained by hearing impairment.

#### Patient 4

she had no symptom except slight thoracic deformation when she was studied at 16 months because of the disease in her brother, patient 3. Biochemical investigations detected keratosulfaturia and a very low level of GALNS activity, leading to Morquio A syndrome. She was treated surgically for tibia osteotomy because of an intense genu valgum which avoided her to walk. Then, the clinical course was similar to her brother's. Gradually, facial dysmorphim and disproportional dwarfism developed. At the age of 5 years, radiographs detected truncal dysmorphim and dorsal-lumbar cyphosis with generalized platyspondylia. Intellectually, the patient was normal and doing well at school.

#### Patient 5

This little girl whose parents were first cousin was the first child, after a normal vaginal delivery. At the age of 4 years, she was referred to paediatric Unit of Kairouan Hospital (Central Tunisia) for medical investigation of severe dysmorphic syndrome.

Retrospectively, the onset of the disease could be ascertained to 2 years of age with abnormal prominence of the breastbone. Gradually, valgus deformities of the knees, funnel like chest with carinate pectus developed associated with characteristic facial appearance (broad mouth, small nose) and abnormal flexibility of joints. The Morquio A diagnosis was confirmed by keratansulfaturia and deficient activity of GALNS in leukocytes. By the age of 8 years, compensatory upper thoracal lower thoraco-lumbar scoliosis and cervicothoracal cyphosis developed. They resulted in progressive shortening of the walking distance and fatigue. Skeletal radiographs showed severe dysostosis, generalized platyspondylia, and important anterior hypoplasia of L1. Cervical MRI indicated compression of the medulla.

Aspastic paraplegia occurred by the age of 10 years and the surgeons refused to perform any operation. Now, hepatomegaly, mitral insufficiency and cornal clouding have developed. Her severe handicap avoided her to go to school, although her intelligence was preserved. In addition, very low socio economical conditions result in extremely bad conditions of life for the child and the whole family.

#### Patient 6

She was the third child born to consanguineous family, after a normal vaginal delivery. By the age of 18 months, this child was evaluated for growth failure, feel walking problems and intensive genu valgum at paediatric department of Sidi Bouzid Hospital in central Tunisia. Associated with facial dysmorphism (protrusion of lower facial maxillary) but normal dentition, the trunk was short and pectus carinatum was observed. She was diagnosed as MPS IVA on the basis of clinical analysis and the diagnosis was demonstrated by biochemical analysis (keratansulfaturia and no detectable activity of GALNS in leukocytes).

At the age of 7 years, skeletal X-rays and cervical magnetic resonance imaging (MRI) typically showed acute osseous deformation with dorsal and lumbar cyphosis. The vertebra was markly flattening and the connection between first and second vertebra in the neck was extremely poorly developed with a compression of the bulbospinal region. Her intelligence was normal and she was studying well at the first year of school. The overall physical handicap was severe and she eventually died few days after spinal deformity surgical treatment, during the molecular study.

#### Patient 7

She was the third child born to consanguineous family and she had 2 MPS IVA unaffected brothers. By the age of 3 years, this girl was referred to paediatric unit of Tunis (north of Tunisia). She was evaluated for facial dysmorphism, protrusion, bone dysplasias, short trunk and neck, sternal bulge and disproportional dwarfism. At the age of 7 years, skeletal X-rays and cervical magnetic resonance imaging (MRI) typically showed acute osseous deformation with dorsal and lumbar cyphosis. By the age of 11 years, radiologic examination detected skeleton malformations on cervical rachis (flat cervical vertebra, hypoplasia of the odontoid apophysic second vertebrae), on thorax (oar-appearing ribs) and rachis. Cardiac examination was normal. Her intelligence was normal and she was studying well at school.

## Methods

PCR reactions allow detection of polymorphisms in GALNS gene. The conditions were described previously [8].

For GALNS haplotyping, the mutant and normal alleles were tested for six polymorphisms by PCR sequencing [8]. The first polymorphism is within the fifth intron (IV5+134; CCAAGG [allele A] or CCGAGG [allele a]) [9]. The second polymorphism is located within exon 7 (GCACGC [alleleB] or GCATGC [allele b] [10]. The third polymorphism is positioned within the seventh intron (IVS7nt90; GTAC [allele H] or GAAC [allele h]. The fourth polymorphism is positioned within exon 11 (GTCC [allele C] or GGCC [allele c]). The fifth polymorphism is located within the exon 13 (AAGCCT [allele D] or AGGCCT [allele d] [9]. The sixth polymorphism (CCAG [allele E] or CCGC [allele e] is located within the exon 14 [11].

## Results

Nine GALNS polymorphisms were detected in the 7 studied patients (table 2). Of these polymorphisms, five are within GALNS gene exons. Six polymorphisms (2, 3, 6, 7, 8, and 9) have been previously described and used for linkage analysis in MPS IVA patients and determination of haplotypes.

**Table 2:** GALNS gene polymorphisms in Tunisian patients

Polymorphisms	Patients	Amino acid	Nucléotide	Exon/intron
1	Patient 6	L67M	CTG > ATG	Exon 2

2 ^a^	Patient 1, patient 2, patient 3, patient 4, patient 5, patient 6, patient 7	H236H	CAC > CAT	Exon 7

3 ^a^	Patient 1, patient 2, patient 3, patient 4, patient 5, patient 6, patient 7	E477E	GAG > GAA	Exon 13

4	Patient 7	IVS13+75T-A*	GTG > GAG	Intron 13

5	Patient 7	IVS3+684C-A*	AAC > ACC	Intron 3

6^a^	Patient 1, patient 2, patient 3, patient 4, patient 5, patient 6, patient 7	-	A > G	Intron 5

7 ^a^	Patient 1, patient 2, patient 3, patient 4, patient 5, patient 6, patient 7	-	A > T	Intron 7

8 ^a^	Patient 1, patient 2, patient 3, patient 4, patient 5, patient 6, patient 7	A393S	GCC > TCC	Exon 11

9 ^a^	Patient 1, patient 2, patient 3, patient 4, patient 5, patient 6, patient 7	-	A > G	Exon 14

* Novel polymorphisms; a: polymorphisms used for haplotypes determination

The previous studies [8,12] showed that patients 1, 2, 3, 4 and 5 present the IVS1+1G-A homozygous mutation while patient 6 present the G66R homozygous mutation.

Haplotypes analysis revealed that IVS1+1G-A mutation occurred in the haplotype A/B/c/d/e/H. The haplotype associated with the G66R mutation was A/b/c/D/e/h. The haplotype detected within patient 7 with A85T mutation was A/b/C/d/E/H.

We have summarized the haplotypes associated with mutations detected within the 7 studied patients in table 3.

**Table 3:** Haplotypes and mutations detected within the studied patients

Patients	Haplotypes	Mutations	
**1**	A/B/c/d/e/H	IVS1+1G-A	Exon 1

**2**	A/B/c/d/e/H	IVS1+1G-A	Exon 1

**3**	A/B/c/d/e/H	IVS1+1G-A	Exon 1

**4**	A/B/c/d/e/H	IVS1+1G-A	Exon 1

**5**	A/B/c/d/e/H	IVS1+1G-A	Exon 1

**6**	A/b/c/D/e/h	G66R	Exon 2

**7**	A/b/C/d/E/H	A85T	Exon 3

We have identified two novel heterozygous polymorphisms in intron 13 and intron 3 (IVS13 + 75T-A and IVS3 + 684C-A) within patient 7. These polymorphisms were identified within the normal Tunisian population.

The other polymorphisms identified within patients 1, 2, 3, 4, 5 and 6 were previously reported.

## Discussion

Morquio A disease is due to the lack of N-acetylgalactosamine-6-sulfate-sulfatase. These deficiencies result in a progressive accumulation of the keratane sulfate. This process leads to progressive and chronic course for visceral attacks of the affected organs such as lungs and heart.

The molecular basis of GALNS deficiency leading to the clinical symptoms of Morquio A disease is of particular interest because of its allelic heterogeneity, clinical variability, and the presence of the common mutation characteristic for each ethnic population.

Polymorphisms may be useful for carrier detection and prenatal diagnosis in families whose specific mutations have been identified.

The screening of polymorphisms in GALNS gene revealed that there are at least 16 polymorphisms detected [4]. Here, we describe two novel polymorphisms in intron 13 and intron 3 within patient 7. These polymorphisms are in the heterozygous status.

Associations between a specific mutation and a specific haplotype can be demonstrated in genetic disorders. To understand the origin of a mutation, it is very useful to identify whether a certain recurrent mutation is associated with the same haplotype. A frequent mutation can be explained by the founder effect [13].

Haplotyping of known GALNS polymorphisms revealed that the splice site mutation was on a common background which suggests that these mutant alleles were "identical by descent" and were derived from a common ancestor.

Individuals resulting from a common ancestor are likely to have inherited both copies of the mutated gene and also in the haplotypes which will be transmitted to descent [14] and thus rare genotypes are maintained.

Further screening in Tunisian population of haplotyping data for the splicing mutation IVS1+1G-A should reveal whether this mutation is common in Tunisian population because of a founder effect. Others screening were also necessary for the analysis of the haplotypes identified within patients 6 and 7.

The frequent haplotype identified in normal individuals is a/b/h/c/D/E [13]. This haplotype was not detected within any one from the patients of this study.

The identification of polymorphisms and mutations in the GALNS gene significantly promoted the understanding of correlation with genotype and phenotype within MPS IVA patients.

The identification of polymorphisms within the 7 studied patients may provide means for prenatal diagnosis for MPS IVA families.

## Conclusion

Screening of polymorphisms in GALNS gene and determination of the haplotypes is necessary to search the origin of mutations within MPS IVA patients or to provide means for prenatal diagnosis if the mutation is undefined.

To understand the origin of a mutation, it is very useful to identify whether a certain recurrent mutation is associated with the same haplotype.

## List of abbreviations

MPS IVA: Mucopolysaccharidosis IVA; GALNS: N-acetylgalactosamine-6-sulfate sulfatase; KS: keratane sulfate; C6S: chondroitine-6-sulfate; PCR: polymerase chain reaction.

## Competing interests

The authors declare that they have no competing interests.

## Authors' contributions

SK and LC Have done all the work (PCR, sequencing...) in the laboratory. SF, AM analysis and interpretation of the results. SL has given final approval of the version to be published. All authors read and approved the final manuscript
